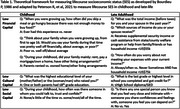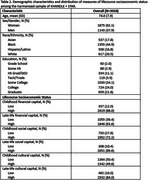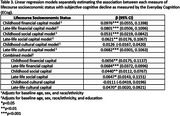# Associations of lifecourse socioeconomic status and subjective cognitive decline as measured by the Everyday Cognition (ECog) among diverse older adults

**DOI:** 10.1002/alz70857_105265

**Published:** 2025-12-25

**Authors:** Nancy X Chen, Rachel Peterson, M. Maria Glymour, Elizabeth Rose Mayeda, Lisa L. Barnes, Paola Gilsanz, Rachel A. Whitmer, Sarah Tomaszewski Farias

**Affiliations:** ^1^ University of California, Davis, Davis, CA, USA; ^2^ University of Montana, Missoula, MT, USA; ^3^ Boston University School of Public Health, Boston, MA, USA; ^4^ UCLA Fielding School of Public Health, University of California, Los Angeles, CA, USA; ^5^ Rush Alzheimer's Disease Center, Rush University Medical Center, Chicago, IL, USA; ^6^ Kaiser Permanente Northern California Division of Research, Pleasanton, CA, USA; ^7^ University of California, Davis School of Medicine, Sacramento, CA, USA

## Abstract

**Background:**

Social determinants of health (SDOH) are the social and economic conditions in which people are born, develop, and age (WHO, 2020). Limited previous work suggest various SDOH may influence subjective cognitive decline (SCD), which is a risk factor for dementia. This study examined associations between lifecourse socioeconomic status (SES) and SCD.

**Method:**

In two multi‐ethnic cohorts of long‐term Kaiser Permanente Northern California members with no prior dementia diagnosis, we examined the association between a set of lifecourse measures of SES and SCD. Drawing from a theoretical framework describing the measurement of lifecourse SES and utilized in our previous work, we constructed variables measuring three aspects of SES (financial, cultural and social domains) based on retrospective ratings of childhood and current SES (Table 1). SCD was measured using the psychometrically validated Everyday Cognition (ECog) scale. Responses, rated 1=“Better or no change” to 4=“Consistently much worse”, were averaged across items. Multivariable linear regression models estimated associations of each lifecourse SES measure with SCD, separately. A combined model including all lifecourse SES measures evaluated independent effects. Models adjusted for baseline age, sex, and race/ethnicity. Late‐life financial and social capital models additionally adjusted for education.

**Result:**

Participants’ (*N* = 3018) mean age was 74±7.9 years, 62% women, 18% identified as Asian, 45% as Black, 17% as Hispanic/Latino, 21% as White, and 16% had ≤high school diploma/GED (Table 2). Low levels of childhood and late‐life financial capital, childhood and late‐life social capital, and late‐life cultural capital were all associated with higher ratings of SCD (b_CFC_=0.09, 95%CI 0.05,0.14; b_LFC_=0.08, 95%CI 0.05,0.11; b_CSC_=0.05, 95%CI 0.02,0.08; b_LSC_=0.06, 95%CI 0.02,0.11, b_LCC_=0.07, 95%CI 0.03,0.11; Table 3). In the combined model, low childhood and late‐life financial capital, childhood and late‐life social capital, and late‐life cultural capital remained significantly associated with worse ECog (Table 3).

**Conclusion:**

Financial, social, and cultural capital across early and late life, play a critical role in shaping SCD. These findings emphasize the importance of addressing socioeconomic inequities in access to resources and support throughout the lifecourse to mitigate SCD, which is a risk factor for the development of cognitive impairment and dementia.